# Aging Impact in Response to Different Classes of Biological Treatment in Psoriatic Patients: A Real-Life Observational Study

**DOI:** 10.3390/jcm12237215

**Published:** 2023-11-21

**Authors:** Francois Rosset, Luca Mastorino, Paolo Dapavo, Michela Ortoncelli, Pietro Quaglino, Simone Ribero

**Affiliations:** Section of Dermatology, Department of Medical Sciences, University of Turin, 10126 Turin, Italy; lucamastorino02@gmail.com (L.M.); paolodapavo@gmail.com (P.D.); mortoncelli@cittadellasalute.to.it (M.O.); pietro.quaglino@unito.it (P.Q.)

**Keywords:** psoriasis, psoriasis and age, age and biological treatment, anti IL-17, anti-IL-23, quality of life, psoriasis comorbidities

## Abstract

Over the last decade, the treatment landscape for moderate to severe psoriasis has undergone transformative changes with the advent of biotechnological drugs. Monoclonal antibodies targeting the IL-17 and IL-23 pathways have displayed remarkable clinical efficacy and safety, even among patients with complex comorbidities. These innovations have extended across various age groups within the psoriatic population. However, a scarcity of age-specific data remains regarding the efficacy and safety of these medications. Our study tries to bridge this gap by systematically presenting data obtained from the analysis of 1055 patients treated for psoriasis with anti-IL17 and anti-IL23 drugs during a 1-year period. The effectiveness and safety of anti-IL-17 and anti-IL23 drugs for moderate to severe psoriasis were assessed across four different age groups ranging from patients less than 26 years old to patients older than 65 years, divided in four year ranges. In the studied population, baseline PASI score was significantly higher in the age group of individuals over 65 years compared to those under 26 years old. Patients over 65 years also exhibited a slower rate of improvement in PASI-90 and PASI < 3 at the 16-week mark compared to other age groups. However, no clinically significant differences in treatment response were found when comparing overall responses among different age groups. In age groups older than 26 years, anti-IL17 drugs seems faster in the achievement of PASI-100 when compared to anti-IL23 drugs. This trend became more pronounced with increasing age. The investigation provides insights into treatment responses and patient characteristics, highlighting the influence of age as a significant variable in patient management.

## 1. Introduction

Psoriasis, a chronic inflammatory skin disorder, encompasses various forms, ranging from mild to severe, with manifestations like plaque psoriasis, guttate psoriasis, pustular psoriasis, inverse psoriasis, and erythrodermic psoriasis [1,2]. In the United States, psoriasis affects approximately 3.2% of adults and 0.13% of children. Worldwide, approximately 125 million people have psoriasis, and prevalence ranges from 0.5% in Asia to 8% in Norway [1]. Women and men are usually affected equally. While psoriasis can manifest at any age, a bimodal age distribution exists for psoriasis presentation at ages 18 to 39 years and also at ages 50 to 69 years [1]. Psoriasis traditionally involves extensor areas of the body, but in its polymorph variant, it can also affect intertriginous areas, as well as the scalp, nails, genitals, and palmoplantar region. The disease manifests as erythematous, shiny, scaly, well-demarcated, and itchy plaques. 

Quantifying the disease is possible in terms of severity and extension using the Psoriasis Area Severity Index (PASI) or the Physician Global Assessment (PGA-g), a 6-point score from 0 (clear skin) to 5 (severe psoriasis) and which only provides information on the severity of the lesion and not its extent [1,2].

These conditions often result from dysregulated immune responses and the overproduction of proinflammatory cytokines, including IL-17 and IL-23 [1,2]. Psoriasis exhibits a broad spectrum of clinical manifestations, and its pathogenic basis may indeed diverge from one patient to another, influenced by genetic predisposition and various environmental factors [1,2]. Mild forms of psoriasis can benefit from topical treatment options including topical corticosteroids, vitamin D analogues, calcineurin inhibitors, keratolytics, and targeted phototherapy [1]. Moderate to severe forms can benefit of phototherapy (narrow-band UVB, or PUVA) and traditional systemic treatment such as metothrexate, cyclosporin, and acitretin [1]. Over the past decade, the landscape of psoriasis treatment has been profoundly transformed by the introduction of biotechnological drugs, specifically monoclonal antibodies targeting the inflammatory pathways of IL-17 (Interleukin-17) and IL-23 (Interleukin-23) [1]. The IL-17 and IL-23 inhibitors currently approved in Europe and Italy are secukinumab, ixekizumab, brodalumab, bimekizumab, risankizumab, guselkumab, and tildrakizumab.

These drugs have exhibited remarkable clinical efficacy, offering a superior benefit–risk profile, even for patients with complex conditions [3] or comorbidities [4,5]. As a result, these biotechnological treatments have become widely accessible, benefiting the majority of the psoriatic population struggling with moderate to severe forms of the disease, regardless of age [6,7]. Biological therapies, in particular anti-IL23 and IL-17 allow the achievement of clear or almost clear skin response, evaluated as reduction of 90% or 100% in the PASI (PASI90 and 100). Despite these achievements, some patients experience biological treatment failure and switch from one treatment to another [1]. The high effectiveness of IL-17 and IL-23 blocking agents in diverse psoriasis populations can be attributed to their highly specific and finely tuned modulation of intricate inflammatory cascades implicated in the pathophysiology of the condition. This modulation is of high significance, particularly considering the potential inter-individual variation in the underlying molecular etiology of psoriasis [6,7]. 

These variations make it mandatory to tailor treatment regimens and therapeutic expectations in accordance with the intricate biological diversity observed in psoriatic patients, thereby optimizing treatment outcomes and fostering the delivery of precision care within this medically and genetically intricate landscape [8,9]. However, when ages are a variable, information regarding the use of these medications in different years groups remains limited and has been derived from a small number of real-world studies [10,11,12]. Furthermore, existing literature tends to evaluate the efficacy of these drugs within isolated age groups [13], such as elderly patients with comorbidities [10,11,12]. Consequently, in our objective to provide a comprehensive understanding of the impact of monoclonal drugs in the management of moderate to severe psoriasis, we tried to understand the effectiveness of biological therapies across different age demographics. Such insights can help healthcare providers tailor treatment strategies more effectively, considering the unique clinical needs and potential challenges faced by patients in each age bracket. 

## 2. Material and Methods

### 2.1. Study Design

This study was designed to investigate the age-stratified response to novel biologic agents in cohorts of psoriatic patients. An observational investigation was conducted at the University Dermatology Clinic of the University of Turin, with the primary objective of systematically assessing the therapeutic effectiveness, tolerability, and clinical outcomes associated with these innovative biological therapies. 

### 2.2. Study Population

The study cohort enrolled all patients >18 years old for whom comprehensive medical record data were retrievable and who had received a minimum of one therapeutic dosage of IL-17 or IL-23 inhibitors within the timeframe spanning from December 2019 to December 2022. These patients were stratified into four distinct age categories: those aged less than 26 years, individuals falling within the 26–40 years age bracket, patients in the 41–65 years age range, and those aged greater than 65 years; this enabled a precise age-wise evaluation of their clinical profiles and therapeutic responses. 

Variables considered included sex, the presence of obesity, body mass index (BMI), diabetes, cardiovascular comorbidities, involvement of challenging-to-treat anatomical sites, and the presence of psoriatic arthropathy. Additionally, factors pertaining to the administered biological drugs, such as drug type, therapeutic response, prior treatment history (naive/multifailure status), instances of treatment suspension, and Psoriasis Area Severity Index (PASI) scores at distinct time points, specifically at 16, 28, and 52 weeks, were scrutinized in detail. These comprehensive datasets were scrutinized for trends and patterns, both in the aggregate dataset and upon further division, specifically segregating the patient cohort according to the subcategories of anti-IL23 and anti-IL17 drug types.

### 2.3. Biological Therapies Considered

We considered in the study the following treatment given as the traditional approved regimen after initial specific induction (please refer to specific datasheet):

- Ixekizumab 80 mg one subcutaneous (SC) injection every 4 weeks;
- Secukinumab 150 mg 2 sc injections every 4 weeks;
- Brodalumab 210 mg 1 sc injection every 2 weeks;
- Guselkumab 100 mg 1 sc injection every 8 weeks;
- Risankizumab 75 mg 2 sc injection every 12 weeks (150 mg fl not available at the time of the study);
- Tildrakizumab 100 mg 1 sc injection every 12 weeks.

### 2.4. Statistical Analysis

Non-parametric tests, specifically the Mann–Whitney U and *t*-Student tests, were systematically applied to assess dichotomous continuous variables. Additionally, for the categorical variables, the Chi-square and Fisher tests were utilized, with the selection between these two tests contingent upon the specific number of patients involved in each analytical comparison. Statistical significance was considered by a *p*-value of less than 0.05.

## 3. Results

In total, 1055 patients were enrolled in the study, of which 61.23% (n = 646) were treated with anti-IL17 while 38.77% (n = 409) were treated with anti-Il23. The first subgroup comprised patients under 26 years old (n = 40), of which 37.5% (n = 15) were treated with anti-IL17 and 62,5% (n = 25) treated with anti-IL23. The second subgroup included patients between 26 and 40 years old (n = 161), of which 55.9% (n = 90) received anti-IL17 and 44.1% (n = 71) received anti-IL23. The third subgroup consisted of patients between 40 and 65 years old (n = 576), of which 63.5% (n = 366) were treated with anti-IL17 and 36.5% (n = 210) with anti-IL23. Finally, the fourth subgroup comprised patients over 65 years old (n = 278), with 62.9 (n = 175) receiving anti-IL17 and 37.1% (n = 103) receiving anti-IL23.

Patient gender distribution among age groups was not significantly differently distributed (Table 1). DLQI (Dermatology Life Quality Index) values did not differ based on age at the beginning of treatment. The BMI was higher in age groups over 40 years compared to those under 40 years (*p* < 0.001, Table 1).

The younger age groups (under 40 years) had a higher representation of psoriasis in difficult-to-treat sites (palms and scalp) (*p* = 0.032, Table 1). Conversely, age groups over 40 years had a higher incidence of psoriatic arthritis (*p* < 0.001, Table 1) compared to patients younger than 40 years old. No cases of psoriatic arthritis were observed in the population under 26 years old.

Diabetes and cardiovascular comorbidities were more prevalent in the older population (*p* = 0.006, Table 1).

Younger patients (namely under 40 years) had a higher percentage of bio-naive patients (*p* = 0.008), while multifailure patients were more represented among the older group (over 40 years) (*p* = 0.044, Table 2). The discontinuation rate of biological drug treatment was similar among the different age groups (*p* = 0.259).

The use of anti-IL17 drugs was significantly higher in patients older than 40 years old (*p* < 0.005, Table 2), while anti-IL23 drugs were more common in patients under 40 years of age, and especially in those under 26 (*p* < 0.001, Table 2). Individual analysis revealed statistically relevant correlations of single molecules with age groups (*p* < 0.001, Table 2): Secukinumab was administered more in older (over 40 years old) age groups, Guselkumab mainly to patients under 40, Brodalumab and Risankizumab to younger age groups, Ixekizumab to older and middle age groups (>40 years), and Tildrakizumab had a peculiar trend, being used mostly in extreme age groups.

In assessing disease progression, factors like absolute PASI and PASI-100, PASI-90, and PASI < 3 at weeks 16, 28, and 52 were considered. The baseline PASI was significantly higher in the over 65 age group compared to the under 26 group (*p* = 0.018). The 40–26 age group had a significantly higher average PASI (*p* = 0.03) than the under 26 group. Patients over 65 had higher average PASI scores at weeks 16 and 28 compared to younger age groups (Table 3).

At 52 weeks, patients across all age groups exhibited similar percentages in reaching PASI-100, except for individuals over 65 who demonstrated comparatively less improvement (*p* = 0.01, Table 3); likewise, for PASI-90, the over 65 age group displayed slower improvement at 16 weeks relative to other age groups (*p* = 0.029, Table 3); similarly, in terms of PASI < 3, the over 65 age group exhibited slower improvement at 16 weeks, particularly when compared to the 40–26 age group (*p* = 0.004, Table 3).

**Figure 1:**
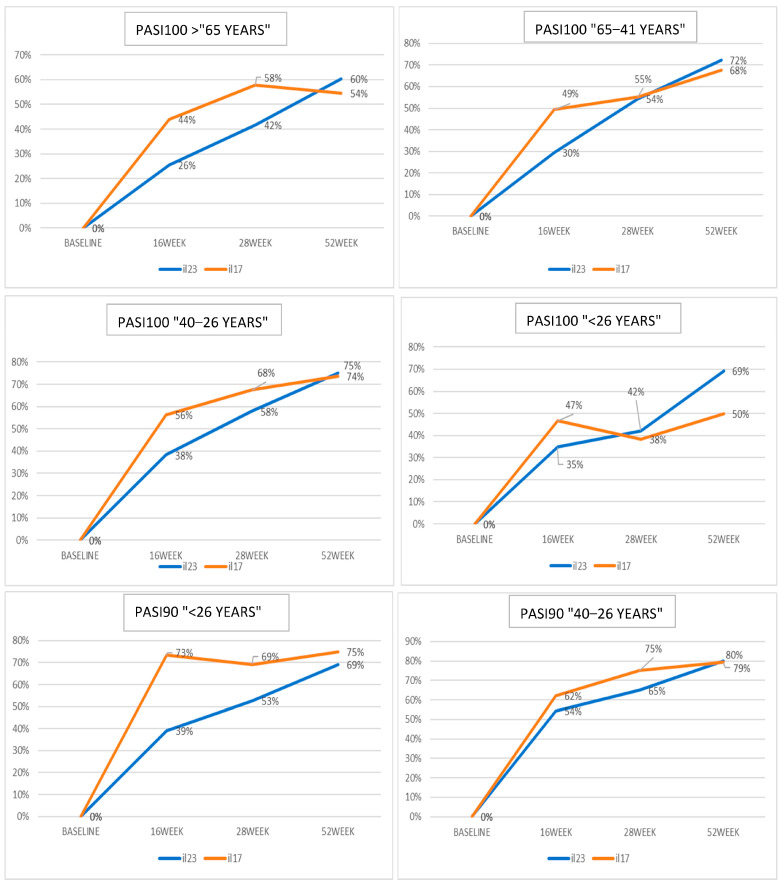

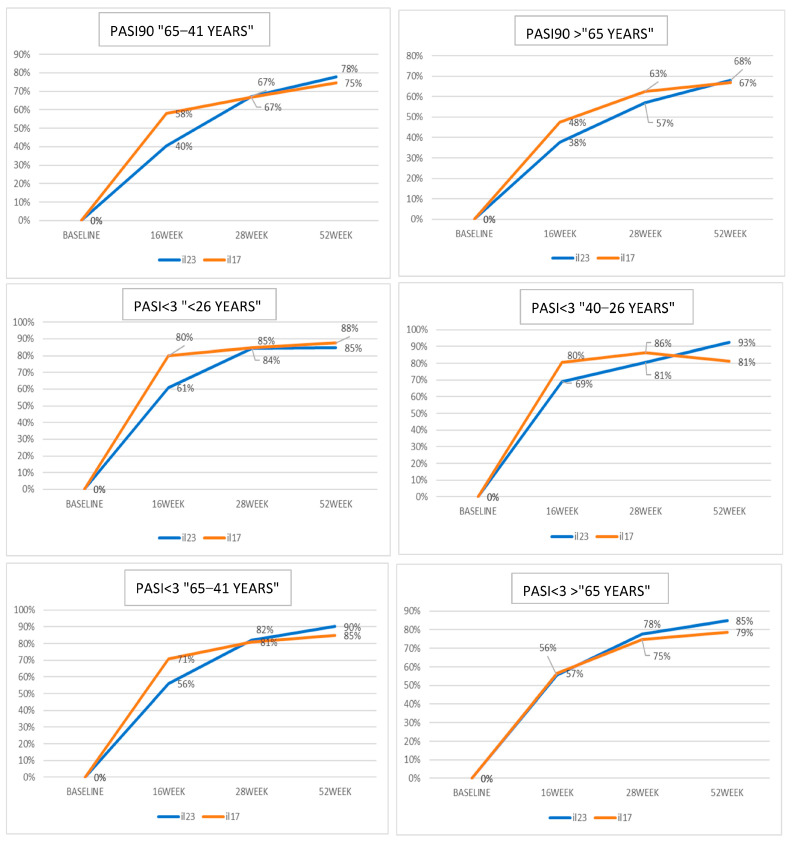
The different diagrams plot the variables concerning response to biological treatments (PASI-100, PASI-90, and PASI < 3) in different age groups. On the y-axis, numbers indicate the proportion of patients that have reached a specific response. Orange lines: anti-IL17 groups; blue lines: anti-IL23 groups.

Considering differences among treatment classes in different age groups, patients over 65 years showed a better treatment response between 16 and 28 weeks in the anti-IL17 class compared to the anti-IL23 class, with a reversal in treatment response at 52 weeks compared to other age groups (Figure 1). The PASI-90 response was similar between groups (Figure 1).

In the 41–65 age group, a statistically significant and rapid response to IL-17 was observed at 16 weeks for PASI-100, PASI-90, and PASI < 3, followed by a subsequent recovery in response to IL-23, while in the 26–40 age group, IL-17 demonstrated superiority for PASI-100, with no significant differences observed for PASI-90 and PASI < 3; moreover, a gradual increase in response to IL-23 was noted, eventually surpassing IL-17 at 52 weeks for PASI < 3 (Figure 1).

No statistically significant differences were found for various time points in the under 26 age group (Figure 1). In this group, IL-17 showed rapid action up to 16 weeks, with a decrease in effectiveness afterward, potentially due to the low sample size.

## 4. Discussion

Our study provides valuable insights into the characteristics and treatment response of psoriatic patients undergoing treatment with new monoclonal drugs. This sheds light on the impact of age as a variable to consider in patient management. Notably, there is no previous data available regarding differences in biologic treatment response rates based on age in psoriatic patients [13]. The few studies present in the literature that explore the association between age and biologic treatments mainly focus on specific age ranges, lacking a comprehensive overview [12,13,14,15,16,17]. In contrast to these studies, our analysis allowed for the examination of multiple age intervals within the same patient population.

The analysis we conducted showed no significant variations in gender distribution or DLQI scores across different age groups. However, it was observed that individuals aged 40 years and above exhibited a higher BMI, which correlates with the increased prevalence of obesity in older age groups [18].

From the analysis, we highlighted a greater prevalence of psoriasis in hard-to-treat areas like the palms and scalp among the younger population. This observation aligns with epidemiological studies on psoriasis [19], suggesting that initiating biological drug therapy early may enhance the effectiveness of managing these problematic regions. On the other hand, older age groups exhibited a heightened occurrence of psoriatic arthritis, along with additional comorbidities such as diabetes and cardiovascular conditions. These findings likely result from prolonged systemic inflammation, which persisted until the introduction of new biologic treatments. This emphasizes the importance of highly effective therapies like biologic drugs not only in treating the skin condition but also its associated comorbidities. This observation is in line with other real-life studies conducted on the elderly population [16,20,21,22,23].

The use of anti-IL17 and anti-IL23 drugs varied among different age groups. Interestingly, anti-IL17 drugs were more frequently prescribed for older individuals, while anti-IL23 drugs were more prevalent in younger demographics. This preference could reflect the desire for better treatment adherence and potential disease-modifying effects offered by anti-IL23 agents [24,25]. Additionally, the preference for treatments requiring fewer injections per year could influence the choice of anti-IL23 drugs, especially among younger, actively employed patients [24]. Furthermore, the selection of specific monoclonal drugs also exhibited variations within each age group, with certain drugs being administered more frequently based on specific age categories.

When evaluating disease progression in response to therapy, the study considered various factors including the absolute Psoriasis Area and Severity Index (PASI), as well as measurements like PASI-100, PASI-90, and PASI < 3 at different time intervals. It was observed that the baseline PASI score was significantly higher in the age group of individuals over 65 years compared to those under 26 years old. Patients over 65 years also exhibited a slower rate of improvement in PASI-90 and PASI < 3 at the 16-week mark compared to other age groups. However, no clinically significant differences in treatment response were found when comparing overall responses among different age groups.

From the analysis of response rates to monoclonal drug therapy, it was evident that in age groups older than 26 years, the speed of response and achievement of PASI-100 were faster with anti-IL17 drugs compared to anti-IL23 drugs. This trend became more pronounced with increasing age over the weeks. In both drug classes, a similar long-term response rate was observed, with the anti-IL23 class showing a recovery of response, consistent with current literature [26,27]. However, in the group of patients under 26 years old, the greater speed of action of IL-17 was not statistically significant due to the limited sample size (n = 15) of patients treated with anti-IL17 in this age group. The lower number of young patients treated with anti-IL17 was primarily due to clinical choice, as the more convenient administration of anti-IL23 better suited the dynamic lifestyle of the younger population compared to adults [24,28].

Immunogenically, the difference in time response is strictly bound with the targeted interleukin pathway [1]: IL-23 primarily plays a pivotal role in orchestrating the inflammatory cascade by stimulating IL-17 production, contributing to the characteristic skin inflammation seen in psoriasis [2]. The rapid response observed with anti-IL-17 treatment may be attributed to the direct inhibition of this proinflammatory cytokine, curbing the acute immune dysregulation [1]. In contrast, anti-IL-23 therapy may exert a more consistent and sustained treatment response by targeting the upstream driver of IL-17 production, thereby modulating the immune response at a fundamental level [1]. Importantly, regulatory T cells (Tregs) are key players in maintaining immune homeostasis and suppressing excessive inflammation. An exploration of the dynamics of Tregs in response to these treatments can offer insights into the observed variations in treatment efficacy [2,22]. A comprehensive grasp of the temporal dynamics of the immune response, with particular attention to the intricate balance between proinflammatory IL-17 and the regulatory role of T regulatory cells (Tregs), is of paramount importance in unraveling the intricate and evolving interplay between these therapeutic biological agents and the complex immunological milieu characterizing psoriasis. The post-treatment evolution of this delicate equilibrium, which hinges on the dynamic shifts in cytokine profiles and immunoregulatory mechanisms, holds significant clinical and therapeutic implications. This profound understanding of the temporal changes in the immune response not only sheds light on the effectiveness and durability of biological interventions but also informs the development of tailored treatment strategies that can harness the immunological evolution to optimize psoriasis management. Such insights may pave the way for novel immunomodulatory approaches aimed at restoring a harmonious balance in the immune system, ultimately yielding improved long-term outcomes for individuals affected by this chronic dermatological condition [9,22]. This knowledge holds promise for further refining therapeutic approaches for this complex skin disorder.

In conclusion, our study has demonstrated that chronological age is not a critical factor in the selection of monoclonal drugs for psoriasis treatment. However, considering age and its associated social factors (such as lifestyle dynamics) and comorbidities [14] can be of significant utility in the clinical decision-making process [24]. This is particularly relevant when choosing between an anti-IL17 or anti-IL23 drug, as it can enhance patient compliance and contribute to improved clinical outcomes and quality of life [21,24].

## Figures and Tables

**Table 1:** Different general variables (sex, diabetes, difficult site, cardiovascular comorbidities, obesity, BMI, DLQI at start and associated arthropathy) are plotted relating with different age groups. In bold significant value (*p* < 0.05).

**Sex**				**Diabetes**				
** Age Group **	** Female **	** Male **	***p***	** Age Group **	** No **	** Yes **	***p***	
>65	38.13%	61.87%	0.06	>65	74.32%	25.68%	**<0.001**	
65–41	31.25%	68.75%		65–41	90.94%	9.06%		
40–26	40.99%	59.01%		40–26	99.27%	0.73%		
<26	37.5%	62.5%		<26	100%	0%		
**Difficult site**				**Cardiovascular comorbidities**				
Age group	No	Yes	*p*	Age group	No	Yes	*p*	
>65	31.14%	68.86%	0.06	>65	11.82%	46.32%	**<0.001**	
65–41	20.28%	79.72%		65–41	58.91%	51.31%		
40–26	15.19%	84.81%		40–26	23.83%	2.38%		
<26	10.26%	89.74%		<26	5.44%	0%		
**Obesity**				**BMI**				
Age group	No	Yes	*p*	Year group	Mean BMI	BMI difference between age groups		* p *
>65	73.83%	26.17%	**0.006**	>65	27.82	>65	65–41	1
65–41	75%	25%		65–41	27.52	>65	40–26	** <0.001 **
40–26	86.54%	13.46%		40–26	24.91	>65	<26	** 0.002 **
<26	86.49%	13.51%		<26	24.29	65–41	40–26	** <0.001 **
				Total	27.07	65–41	<26	** 0.004 **
						40–26	<26	1
**DLQI at start**				**Associated arthropathy**				
Age group	Mean	Between vs. within groups (*p* value)		Age group		No	Yes	***p*** **< 0.001**
>65	22.57	0.58		>65		71.58%	28.42%	
65–41	22.67			65–41		69.44%	30.56%	
40–26	22.93			40–26		82.61%	17.39%	
<26	24.45			<26		100%	0%	
All ages	22.74							

**Table 2:** Different variables related to the biological therapy (naïve for biologics, multifailure for biologic drug, treatment interruption, type of interleukin inhibitors, drug distribution) are plotted in relation to different age groups. In bold significant value (*p* < 0.05).

**Naive for Biologic**				**Multifailure for Biologic Drug**			
** Age Group **	** No **	** Yes **	***p***	** Age Group **	** No **	** Yes **	***p***
>65	41.37%	58.63%	**0.008**	>65	97.84%	2.16%	**0.044**
65–41	38.02%	61.98%		65–41	99.48%	0.52%	
40–26	27.95%	72.05%		40–26	100%	0%	
<26	22.5%	77.5%		<26	100%	0%	
**Treatment interruption**				**Type of interleukin inhibitors**			
Age group	No	Yes	*p*	Age group	IL-17	IL-23	*p*
>65	77.34%	22.66%	0.259	>65	62.95%	37.05%	**0.005**
65–41	81.08%	18.92%		65–41	63.54%	36.46%	
40–26	84.47%	15.53%		40–26	55.9%	44.1%	
<26	85%	15%		<26	37.5%	62.5%	
**Drug distribution**							
Age group	Risankizumab	Guselkumab	Secukinumab	Brodalumab	Tildrakizumab	Ixekizumab	*p*
>65	17.63%	2.88%	25.9%	19.42%	16.55%	17.63%	**<0.001**
65–41	22.92%	6.6%	26.56%	17.19%	6.94%	19.79%	
40–26	27.33%	11.8%	16.77%	24.22%	4.97%	14.91%	
<26	27.5%	22.5%	7.5%	25%	12.5%	5%	

**Table 3:** Different variables of response to biological therapy (PASI, PASI-100, PASI-90 and PASI < 3) at weeks 0, 16, 28, and 52 are plotted relating with different groups age. In bold significant value (*p* < 0.05).

**PASI at Start**						**PASI 16 Weeks**					
** Age Group **	** Mean **	** Differences between Age Groups **		*** p ***		** Age Group **	** Mean **	** Differences between Age Groups **		*** p ***	
** >65 **	** 15.07 **	>65	65–41	1		>65	3.35	>65	65–41	** 0.042 **	
65–41	14.55	>65	40–26	1		65–41	2.5	>65	40–26	** 0.001 **	
40–26	15.03	>65	<26	** 0.018 **		40–26	1.78	>65	<26	0.31	
<26	11.73	65–41	40–26	1		<26	1.95	65–41	40–26	0.36	
All ages	14.65	65–41	<26	0.057		All ages	2.58	65–41	<26	1	
		40–26	<26	** 0.03 **				40–26	<26	1	
**PASI 28 weeks**						**PASI 52 weeks**					
Age group	Mean	Differences between age groups		* p *		Age group	Mean	Differences between age groups		* p *	
>65	2.18	>65	65–41	** 0.028 **		>65	1.49	>65	65–41	0.375	
65–41	1.46	>65	40–26	** 0.002 **		65–41	1.16	>65	40–26	0.433	
40–26	0.96	>65	<26	0.945		40–26	0.95	>65	<26	0.635	
<26	1.34	65–41	40–26	0.575		<26	0.81	65–41	40–26	0.248	
All ages	1.56	65–41	<26	1		All ages	1.2	65–41	<26	0.732	
		40–26	<26	1				40–26	<26	0.267	
**PASI-100 16 weeks**				**PASI-90 16 weeks**				**PASI < 3 16 weeks**			
Age group	No	Yes	*p*	Age group	No	Yes	*p*	Age group	No	Yes	*p*
>65	159	95	0.151	>65	142	112	0.029	>65	106	148	**0.004**
65–41	323	236		65–41	270	289		65–41	192	367	
40–26	80	76		40–26	64	92		40–26	38	118	
<26	24	15		<26	18	21		<26	12	27	
**PASI-100 28 weeks**				**PASI-90 28 weeks**				**PASI < 3 28 weeks**			
Age group	No	Yes	*p*	Age group	No	Yes	*p*	Age group	No	Yes	*p*
>65	104	115	0.057	>65	86	133	0.164	>65	53	166	0.198
65–41	226	275		65–41	165	336		65–41	94	407	
40–26	50	88		40–26	40	98		40–26	22	116	
<26	19	13		<26	13	19		<26	5	27	
**PASI-100 52 weeks**				**PASI-90 52 weeks**				**PASI < 3 52 weeks**			
Age group	No	Yes	*p*	Age group	No	Yes	*p*	Age group	No	Yes	*p*
>65	72	93	**0.01**	>65	54	111	0.119	>65	32	133	0.373
65–41	116	259		65–41	92	283		65–41	51	324	
40–26	24	69		40–26	19	74		40–26	13	80	
<26	8	13		<26	6	15		<26	3	18	

## Data Availability

Data available upon reasonable request.
